# The current status of women’s health nursing in the United States

**DOI:** 10.4069/whn.2024.08.20.1

**Published:** 2024-09-30

**Authors:** Eun-Ok Im

**Affiliations:** School of Nursing, The University of Texas at Austin, TX, USA

## Introduction

With recent political developments in the United States (U.S.), such as the Supreme Court’s decision to overturn *Roe v. Wade* [1], healthcare providers, including nurses, are increasingly concerned about the current state of women’s health. These political events have had a significant impact on the field. Numerous nursing professional organizations have released position statements addressing these issues. For example, the American Academy of Nursing recently published a position statement on access to sexual and reproductive health care [2]. Furthermore, challenges specific to the nursing profession, such as nurse shortages [3] and shortages of nursing faculty [4], are also affecting women’s health nursing in the U.S. Additionally, the recent Future of Nursing 2020–2030 report by the National Academy of Medicine (NAM) [5] and the American Association of Colleges of Nursing (AACN) New Essentials [6] have led to curriculum changes across all nursing programs in the U.S. Consequently, women’s health nursing in the U.S. is undergoing a variety of both expected and unexpected changes in all aspects.

The purpose of this paper is to discuss the current status of women’s health nursing in the U.S. Initially, it describes the sociopolitical and academic contexts of women’s health nursing in the U.S. Subsequently, it addresses the current issues in women’s health nursing within the realms of education, practice, and research. Finally, the paper offers suggestions for the future of women’s health nursing.

## The context of women’s health nursing in the United States

Up until the early 1990s, the U.S. did not experience the severe nurse shortages that are witnessed today. During the 1990s, hospital restructuring and downsizing led to numerous reports of hiring freezes and layoffs of registered nurses (RNs) [7]. Many nurses were deeply concerned about their physical and economic well-being, as well as the quality of patient care provided in hospitals [7]. As a result of these restructurings and downsizings, hospitals often replaced RN staff with nursing assistants who were both poorly trained and poorly paid [8]. During this period, many displaced nurses transitioned their careers to different fields, including the booming IT industry. Over the next 20 to 30 years, this shift greatly contributed to a significant shortage of RNs, with a serious impact on the U.S. healthcare system.

Following seminal reports that highlighted lower patient morbidity and mortality rates in hospitals with a higher proportion of RNs [9-11], hospitals recognized the critical role of RNs in maintaining high-quality care and began to actively recruit them once more. However, nurses who had transitioned to other fields were reluctant to return to bedside care, and the existing nursing workforce was aging, with an increasing number of nurses retiring, exacerbating the shortage of RNs. Additionally, the new generation of nurses places a high value on work-life balance, further complicating efforts to recruit sufficient staff to address the shortfall. Consequently, the nurse shortage in the U.S. is expected to be prolonged [12].

Currently, the U.S. has fewer than 10 nurses per 1,000 population [13]. Very few states have between 10 and 12 nurses per 1,000 population, and only three states report having between 12 and 16 nurses per 1,000 population (Table 1). The most severe shortage is in Utah, with only six nurses per 1,000 population. Texas follows with seven nurses per 1,000 population, while New York has nine nurses per 1,000 population. Washington DC, in contrast, boasts the highest ratio, with more than 17 nurses per 1,000 population. According to the U.S. News and World Report, although the demand for RNs in the U.S. has been increasing steadily in a linear pattern, the number of employed RNs has fluctuated, particularly dipping in 2020 due to coronavirus disease 2019 (COVID-19) [14]. Despite a slow recovery since then, the gap remains.

The situation among nursing faculty is similarly dismal. In recent years, virtually all mid-level and top-tier nursing schools in the U.S. have increasingly hired faculty members from outside the country. Specifically, the number of Korean nurse faculty members in the U.S. has grown significantly. In the late 1990s, there were only a few nursing professors of Korean heritage teaching at U.S. nursing schools. However, the Global Korean Nursing Foundation recently estimated that over 100 nursing professors of Korean heritage are currently working across the U.S. (based on per­sonal communication in 2022). Another noticeable trend highlighting the severe lack of nursing faculty in the U.S. is the ease with which Korean Ph.D. graduates, who earn their doctoral degrees in the U.S., can secure faculty positions in the U.S. and choose to stay after completing their degrees. In contrast, it is more challenging for them to obtain faculty positions in South Korea. Most nursing schools in the U.S. are now taking on the additional burden of supporting visas for overseas faculty members to ensure they have an adequate number of faculty members. Indeed, the shortage of nursing faculty has become a severe and inevitable phenomenon in recent years.

## Women’s health nursing: education

The Institute of Medicine (IOM) has played a pivotal role in shaping the trends and policies of nursing education in the U.S. In 2015, the IOM became the NAM, which subsequently issued a new Future of Nursing report following the COVID-19 pandemic [5]. This report from the NAM on the future of nursing merits close examination. It includes significant recommendations such as permanently removing barriers to care, increasing recognition of nurses’ contributions, equipping nurses to achieve health equity, and diversifying the nursing workforce. Despite the importance of these recommendations, they were not universally welcomed within nursing academia. Many nursing schools chose to focus on the New Essentials proposed by the AACN instead [6].

The New Essentials led to a major shift in nursing education and nursing practice by emphasizing competency-based education. This focus has prompted extensive revisions in nursing curricula throughout the U.S., establishing the 10 domains of the New Essentials as foundational elements of nursing education. Notably, some updates to the essentials reflect the evolving healthcare needs in the U.S. For example, influenced largely by the COVID-19 pandemic, a new domain focusing on “quality and safety,” particularly in relation to disaster nursing, has been emphasized in the AACN New Essentials.

A general sentiment was shared between the Future of Nursing report and the AACN New Essentials regarding these focal points, though there were subtle differences. For example, the Future of Nursing report places a strong emphasis on preparing a workforce well-versed in disaster nursing. In contrast, the AACN mentions disaster nursing under the category of “quality and safety.” Another distinction is seen in the treatment of nursing policy making; it was a prominent feature in the Future of Nursing, whereas in the AACN framework, it is incorporated under the domain of “professional leadership development.” Overall, the tone of the Future of Nursing report is relatively stronger than that of the AACN’s New Essentials.

## Women’s health nursing: practice

The women’s health workforce in the U.S. primarily consists of obstetrics and gynecology (OB/GYN) physicians, nurses, and physician assistants (PAs). Within the field of OB/GYN, there is a diverse range of board-certified subspecialists, including gynecologic oncology, female pelvic medicine, reproductive endocrinology and infertility, and maternal-fetal medicine. Consequently, patients must carefully consider their immediate healthcare needs to choose the appropriate provider. Another emerging trend is the growth of hospitalists, which shifts the focus from private practices to care primarily provided in hospital settings by teams of specialists. Additionally, there is an increasing concern over physician burnout and a decline in the number of clinical scientists. This decline is partly attributed to the fact that providers can maintain a comfortable living solely from clinical practice, avoiding the additional pressures associated with research. Technological advancements have also reshaped the practice of women’s health nurses. Hospitals across the U.S. are integrating new technologies such as robots for transporting supplies, robotic surgery, and the widespread adoption of electronic medical records (EMRs). Despite these advancements aimed at enhancing healthcare delivery, the use of EMRs has been found to potentially increase the distance between providers and patients. This change underscores the growing importance of building strong rapport and interpersonal connections, particularly for nurses in women’s health.

To understand the evolving trends, it is worthwhile to review the history of women’s health nursing in the U.S.

### Historical developments in women’s health

The 1830s and 1840s marked the beginning of women’s health nursing in the U.S., coinciding with the first wave of feminism [15]. This era saw the emergence of a movement advocating for women’s equal rights and the transformation of the patriarchal healthcare system to better support female patients, signaling the start of women’s health nursing. It is important to note that some contend the women’s health movement began in the 1890s, driven by Margaret Sanger’s campaign for birth control rights for U.S. women [15].

Some argue that women’s health nursing originated in the 1960s, a time when women’s reproductive rights were restricted and abortion was illegal [15]. During this period, it is estimated that between 500 and 1,000 women died annually from complications related to illegal abortions [16]. The women’s health movement contributed to the Supreme Court’s decision in *Roe v. Wade* in 1973, which legalized women’s reproductive rights [15]. Consequently, in the 1970s, many healthcare providers and women’s health nurses began to advocate for a comprehensive approach to women’s health. By 1973, over 1,200 women’s self-help health groups had formed in the U.S. [17]. These groups advocated for a shift in childbirth practices, moving from a medically interventionist approach to treating childbirth as a normal process. Around the same time, the Lamaze movement and the International Childbirth Education Association were founded, and the involvement of women’s partners in the birthing process was encouraged [15]. Thus, maternity care in traditional hospitals transitioned to family-centered care, which became widely popular in the 1980s. This period also marked the establishment of childbirth classes as essential components of prenatal care.

In the 1980s, the conservative political environment gained momentum with the Reagan administration and the dominance of the Republican Party. During this period, anti-abortion activists began advocating for the repeal of legalized abortion. Consequently, feminist health clinics and fertility control clinics increasingly became targets of attack [15]. By 1989, the Supreme Court started restricting abortions [16].

The 1980s and 1990s marked a significant period for women’s health, particularly following the formal establishment of the Congressional Caucus for Women’s Health Issues in 1977 as a Legislative Service Organization [15]. This development allowed women’s health issues to be addressed in Congress, transcending political affiliations and preferences. The creation of the Task Force on Women’s Health Issues within the U.S. Public Health Service in 1983 led to several key recommendations for women’s health, including the inclusion of both women and men in health research. Historically, women were excluded from health studies under the pretext of protection, which limited the applicability of research findings to women and led to numerous issues. In response, the National Institutes of Health (NIH) implemented a policy in 1986 mandating the inclusion of both women and men in NIH-funded clinical research [18]. Prior to this policy, only 13.5% of NIH grants were focused on women’s health, and women were largely omitted from clinical research [19]. Following this change, the NIH established the Office of Research on Women’s Health (ORWH) in 1990. The ORWH has since been developing women’s health-related agendas and providing trans-institutional funding for women’s health, acting as a supplemental funding source alongside grants funded by individual institutions. The first women’s health study funded through this mechanism was the Women’s Health Initiative study, which secured an investment of 625 million U.S. dollars over the subsequent 14 years [15].

In 1993, a significant milestone was achieved when the U.S. Food and Drug Administration lifted a restriction that had been in place since 1977, which prohibited women from participating in new drug testing [15]. This change enabled a deeper understanding of how gender differences affect medication responses and side effects in women compared to men. The following year, the Centers for Disease Control and Prevention (CDC) made another substantial investment in women’s health by allocating 2 million U.S. dollars for chlamydia screening for women and their partners, and 51 million U.S. dollars for pap smear testing and mammography screening for low-income women [15]. Additionally, the Office of Women’s Health was established within the CDC to provide initiatives (programs and activities) and coordinate policies aimed at improving women’s health.

### Recent developments impacting women’s health

Readers may be familiar with the more recent upheavals since 2020. The Supreme Court’s recent decision to overturn *Roe v. Wade* has effectively reinstated the prohibition of abortions across the U.S. [20]. Currently, abortion access is restricted based on gestational age in 28 states, with 14 states imposing near-total bans. These policies, supported by conservative groups, have significant influences on women’s health. Additionally, the restrictions on women’s health practices have led to a decline in women’s health specialty programs in nursing schools, as practitioners face increasing conflicts.

In 2022, the American Nurses Association (ANA) declared that sexual health is a human right, that abortion is a reproductive health option, and that nurses should have the freedom to discuss these topics with patients to facilitate informed decision-making [21]. This declaration emphasizes the responsibility of nurses to provide information and respect patients’ choices regarding sexual health and pregnancy, highlighting their role in supporting patients. However, nurses often face challenging situations in practice, as they must navigate ethical dilemmas that may conflict with legal constraints. The ANA’s 2022 Position Statement [22] was supported by the Association of Women’s Health, Obstetric and Neonatal Nurses in the same year [23], and later by the American College of Nurse-Midwives in 2024 [24], as well as the National Association of Nurse Practitioners in Women’s Health [25].

The ANA website currently offers guidelines for nurses operating in states with restrictive laws, advising them to carefully consider the ethical and legal complexities involved. For women’s health practitioners in these restrictive states, extreme caution is necessary for conversation and decision. In practice, many women living in restrictive states travel to states with more permissive laws to obtain abortions, including in situations where the pregnant woman’s life is at risk. This travel incurs significant costs and legal risks for the women involved.

## Women’s health nursing: research

For nurse researchers, the NIH, particularly the National Institute of Nursing Research (NINR), represents the primary source of funding. Consequently, research trends have largely aligned with the focal areas emphasized by the NINR. Dr. Patricia A. Grady, PhD, RN, FAAN, who served as the Director of NINR for over two decades and holds a doctorate in physiology from the School of Medicine at the University of Maryland, championed biobehavioral research that emphasized symptom science and palliative care. This influence is evident in the historical scientific priorities of the NINR, which include symptom science, wellness, end-of-life and palliative care, innovation promotion, and self-management. Accordingly, women’s health research has also been guided by these themes. Examples include studies on managing symptoms during the menopausal transition, encouraging physical activity among women, and fostering self-care for various specific health conditions throughout women’s lifespans.

**Figure 1.**
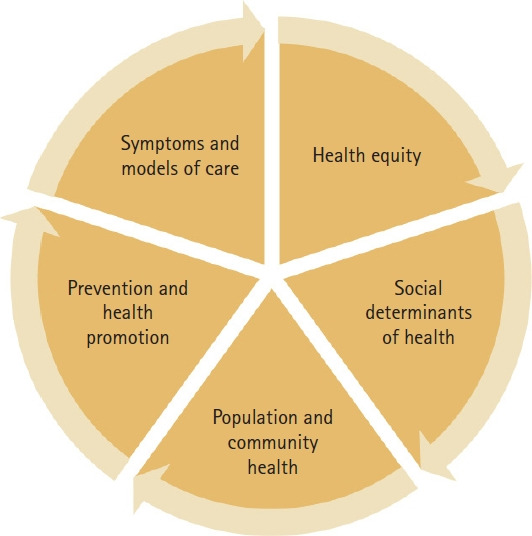
The National Institute of Nursing Research research lenses. This figure is based on information from https://www.ninr.nih.gov/aboutninr/ninr-mission-and-strategic-plan/research-framework.

Since September 2020, Dr. Shannon N. Zenk, PhD, MPH, RN, has served as the director of the NINR. Her research interests in community-based studies and health disparities, coupled with the significant challenges posed by COVID-19 at the time of her appointment, have influenced a shift in the NINR’s focus. The Strategic Plan for 2022-2026, developed over the past 2 years, reflects these changes [26]. Notably, there has been a significant increase in the involvement of ethnic minorities in the NINR strategic planning group. The new Strategic Plan outlines five high-priority research areas: (a) health equity, (b) social determinants of health, (c) population and community health, (d) prevention and health promotion, and (e) systems and models of care (Figure 1). In particular, the addition of “systems and models of care” as the final research lens addresses the nursing workforce issues highlighted by the COVID-19 pandemic.

**Figure 2.**
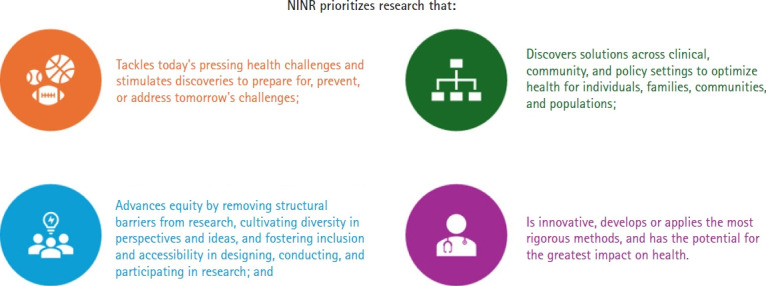
The National Institute of Nursing Research (NINR) guiding principles. This figure is based on information from https://www.ninr.nih.gov/aboutninr/ninr-mission-and-strategic-plan/research-framework.

NINR has also issued guiding principles (Figure 2) that all nurse researchers, including women’s health researchers, must carefully consider when submitting proposals. These principles emphasize the use of innovative and rigorous methods, underscore the importance of achieving the greatest impact on individual, community, and population health, and promote the advancement of equity, diversity, and inclusion. This approach requires not only addressing current pressing health challenges but also focusing on prevention. Researchers are expected to propose solutions that optimize healthcare across clinical, community, and policy settings.

### Trends in women’s health nursing research

Under the significant influence of the recently published NIH and NINR strategic plans, research in women’s health that promotes health equity has seen a marked increase. For example, there has been a rise in the number of NIH-funded studies focusing on racial and ethnic minorities, including immigrant women, rural populations, gender minorities such as the LGBTQ (lesbian, gay, bisexual, transgender, and queer) community, and older adults. Another emerging trend is the growth in NIH-funded community-based participatory research (CBPR) studies. These studies are characterized by their large-scale population-based approach, which ensures meaningful contributions to the community. They also feature active involvement from community gatekeepers and consultants, and direct engagement of participants as team members. Additionally, since the establishment of the Patient-Centered Outcomes Research Institute in 2010, following *the Patient Protection and Affordable Care Act*, there has been an increased emphasis on CBPR. This shift highlights the growing importance of community-focused research over the past decade. Translation and Implementation research represents another key trend in women’s health. This area focuses on adapting scientific evidence for community use, testing its efficacy, and then implementing it in real-world settings. Finally, technology-based approaches are increasingly prevalent, with developments in telehealth, artificial intelligence, robotics-based interventions, and the continued relevance of biomarkers in women’s health studies.

## Future directions for women’s health nursing

What are the future directions for women’s health nursing? I believe that nursing education for women’s health must first and foremost undergo curricular changes to align with the new competencies mandated by the AACN essentials across various programs. Additionally, nursing education should include content on new technologies and subspecialties that are evident in practice. Given the multitude of ethical issues within women’s health nursing, this is another critical area for educational focus. Women’s health education also needs to integrate current trends in topics, populations, and methods, particularly at the graduate level. Furthermore, interdisciplinary partnerships in undergraduate education warrant more attention, especially concerning non-nursing preceptors, in light of the general decline in women’s health practitioners and the nursing workforce. Physicians, PAs, and social workers can provide non-traditional practice experiences for nursing students, who in turn can learn about the unique and critical roles these providers play in women’s health.

Effective nursing practice in women’s health requires setting clear goals for the future nursing workforce. This approach will facilitate necessary actions, enable tracking of workforce outcomes, and help develop strategies to attract more nurses to the field of women’s health. Given the potential ethical issues associated with women’s health practice, open discussions about ethical contexts are crucial. These discussions can also inform and guide nursing education. Additionally, establishing funding sources for training more women’s health nurses would significantly strengthen the women’s health workforce.

For nursing research in women’s health, it is imperative to stay informed about shifts in funding priorities, both nationally and internationally. It is equally important to recognize that research needs will evolve with demographic changes, necessitating the adoption of new methodologies and the integration of technological advancements. Building interdisciplinary research teams is vital, as collaboration is key for nurses to advance women’s health research. Additionally, women’s health nurses should actively engage in shaping and supporting policy changes to promote women’s health research. For example, when funding agencies, including the NIH, are developing their strategic plans, women’s health nurses must participate in the strategic planning process to ensure their research needs are adequately reflected in funding priorities. Lastly, there is a pressing need for more studies on the ethical and legal dilemmas in women’s health to provide the necessary evidence and guidance for women’s health practice and education.

## Figures and Tables

**Table 1.** Proportion of registered nurses (RNs) per population in various states in the United States (2022)

State	State population	No. of of RNs	RNs per 1,000 population
USA (Overall)	333,287,557	3,072,670	9.22
District of Columbia	671,803	11,820	17.59
New York	19,677,151	190,470	9.68
Florida	22,244,823	197,630	8.88
California	39,029,342	325,620	8.34
Hawaii	1,440,196	11,800	8.19
Maryland	6,164,660	49,790	8.08
Texas	30,029,572	231,060	7.69
New Mexico	2,113,344	15,910	7.53
Utah	3,380,800	22,830	6.75

Data source: U.S. Bureau of Labor Statistics and U.S. Census Data.
